# *Histoplasma capsulatum* Encodes a Dipeptidyl Peptidase Active against the Mammalian Immunoregulatory Peptide, Substance P

**DOI:** 10.1371/journal.pone.0005281

**Published:** 2009-04-22

**Authors:** Kendal G. Cooper, Robert Zarnowski, Jon P. Woods

**Affiliations:** 1 Department of Medical Microbiology and Immunology, University of Wisconsin, Madison, Wisconsin, United States of America; 2 Department of Biology, University of Texas-Pan American, Edinburg, Texas, United States of America; University of Minnesota, United States of America

## Abstract

The pathogenic fungus *Histoplasma capsulatum* secretes dipeptidyl peptidase (Dpp) IV enzyme activity and has two putative *DPPIV* homologs (*HcDPPIVA* and *HcDPPIVB*). We previously showed that *HcDPPIVB* is the gene responsible for the majority of secreted DppIV activity in *H. capsulatum* culture supernatant, while we could not detect any functional contribution from *HcDPPIVA*. In order to determine whether *HcDPPIVA* encodes a functional DppIV enzyme, we expressed *HcDPPIVA* in *Pichia pastoris* and purified the recombinant protein. The recombinant enzyme cleaved synthetic DppIV substrates and had similar biochemical properties to other described DppIV enzymes, with temperature and pH optima of 42°C and 8, respectively. Recombinant HcDppIVA cleaved the host immunoregulatory peptide substance P, indicating the enzyme has the potential to affect the immune response during infection. Expression of *HcDPPIVA* under heterologous regulatory sequences in *H. capsulatum* resulted in increased secreted DppIV activity, indicating that the encoded protein can be expressed and secreted by its native organism. However, *HcDPPIVA* was not required for virulence in a murine model of histoplasmosis. This work reports a fungal enzyme that can function to cleave the immunomodulatory host peptide substance P.

## Introduction

*Histoplasma capsulatum* is a thermally dimorphic fungal pathogen of humans and other mammals. This ascomycete is found globally in soils, often associated with bird and bat guano, and causes the respiratory disease known as histoplasmosis [1]. Infection begins by inhalation of microconidia or mycelial fragments small enough to be deposited in the lung alveoli. There the mold undergoes a morphogenic transition to the yeast form that survives and replicates within host macrophages. *H. capsulatum* is capable of infecting immunocompetent individuals, but greater morbidity and mortality are observed when cell-mediated immunity is compromised.

Dipeptidyl peptidase type IV (DppIV) enzymes cleave dipeptides from the N-terminus of proteins after a proline or less efficiently alanine in the second position, releasing X-Pro and X-Ala dipeptides. Human CD26 is a DppIV enzyme found in soluble serum and cell surface membrane-bound forms that has diverse functions including immunomodulatory effects. These effects include alteration – usually inactivation – by enzymatic cleavage of several chemokines such as substance P and RANTES, and modulation of T lymphocyte proliferation via interaction with the CD3 signaling pathway and binding of adenosine deaminase (ADA) [2], [3]. Substance P is a neuropeptide and chemokine that affects cellular immune responses and inflammatory granuloma formation [4], [5], which are hallmarks of *H. capsulatum* infection and critical for control of histoplasmosis [1].

DppIV enzymes are widely distributed among microorganisms as well as mammals, with members present in bacteria, fungi and protozoans. The main function of microbial DppIV enzymes is thought to be provision of nutrition to the microorganism in the form of dipeptides. This hypothesis is based on the observation that DppIV- and aminopeptidase-deficient bacteria have a reduced growth rate reversible by the addition of amino acids to the medium [6]. However, DppIV enzymes can also contribute to virulence of some pathogenic microorganisms such as *Porphyromonas gingivalis*. This bacterium encodes a DppIV (PgDppIV) that is localized in the bacterial outer membrane. Mice infected with a *PgDppIV*-null mutant have significantly increased recruitment of inflammatory cells to sites of infection [7]. Purified PgDppIV cleaves N-terminal peptides of certain chemokines *in vitro*, suggesting that inactivation of immunomodulatory chemokines by truncation may be responsible for the reduced influx of inflammatory cells *in vivo* [8]. PgDppIV also contributes to host connective tissue destruction and binds fibronectin, which may aid in colonization [9].

*H. capsulatum* encodes two putative *DPPIV* genes, *HcDPPIVA* and *HcDPPIVB*. We previously showed that although HcDppIVA contains a predicted signal sequence, it does not detectably contribute to secreted DppIV activity in *H. capsulatum* culture supernatants. Instead, *HcDPPIVB* encodes the majority of extracellular DppIV activity under standard laboratory conditions (Cooper *et al.*, submitted). Here, we show that *HcDPPIVA* encodes a functional DppIV enzyme that is also capable of being secreted into *H. capsulatum* culture supernatant when driven under heterologous promoter and terminator sequences. We expressed recombinant HcDppIVA in *Pichia pastoris*, biochemically characterized the enzyme, and determined that it cleaves the host immunoregulatory peptide substance P.

## Methods

### Strains and media

*Histoplasma capsulatum* strains G217B*ura*5-23 and Δ*HcDPPIVA* were used for this study [10] (Cooper *et al.*, submitted). *Pichia pastoris* strain X-33 (Invitrogen, Carlsbad, CA) was used for heterologous expression and purification of recombinant HcDppIVA. *P. pastoris* transformants were selected on yeast extract peptone dextrose medium (YPD) containing 500 µg/mL zeocin. Buffered glycerol-complex medium (BMGY) was used to grow *P. pastoris* in broth, while buffered methanol-complex medium (BMMY) was used to induce expression of *HcDPPIVA*. *Escherichia coli* JM109 grown in Luria-Bertani broth (LB) was used for cloning and propagation of plasmids. *H. capsulatum* was maintained in *Histoplasma* macrophage medium (HMM) as previously described [11]. Plasmids were transformed by electroporation into *H. capsulatum* strains as previously described [11].

### Fungal supernatant preparation

*H. capsulatum* culture supernatants were harvested by pelleting cells at 1200×g for 10 min at 24°C. Supernatants were filtered with 0.22 µm PES membranes and concentrated 15–30× using regenerated cellulose filter devices with a molecular cut-off weight of 5 kDa (Millipore, Bedford, MA). Total protein concentration was determined using the Bradford assay (Bio-Rad, Hercules, CA).

### Microtiter plate DppIV enzymatic assay

90 µl of each supernatant was added to 96 well plates. 10 µl of Gly-pro-7-amido-4-methylcoumarin hydrobromide (Gly-pro-AMC) was added for a final concentration of 200 µM (Sigma, St. Louis, MO). Samples were incubated at 37°C with shaking. Enzymatic cleavage leading to the release of the fluorescent AMC molecule was measured using a microplate spectrofluorometer (SPECTRAmax Gemini EM, Molecular Devices) with excitation and emission wavelengths of 360 and 440 nm, respectively.

### In-gel zymogram DppIV enzymatic assay

Equal amounts of supernatant protein were separated on 6% native PAGE gels containing no SDS. After electrophoresis, gels were overlaid with 0.3% agarose in 50 mM Tris pH 8 containing 400 µM Gly-pro-AMC and incubated at 37°C for 20 minutes. Bands of fluorescence were visualized using a Gel Doc 1000 documentation system (Bio-Rad). Gels were subsequently stained with Coomassie Brilliant Blue to visual total protein.

### Northern hybridization

RNA was collected from late log phase cultures of G217B using RiboPure kit (Ambion) following manufacturer's instructions. 23–28 µg RNA was electrophoresed in a 1% formaldehyde agarose gel and then transferred to Hybond N+ nylon transfer membrane (Amersham). The blot was probed with a labeled plasmid containing *HcDPPIVA*. The blot was then stripped by boiling in 0.5% SDS and re-probed with a labeled plasmid containing 590 bp of *H. capsulatum ACT1* cDNA for normalization with the actin gene transcript.

### Preparation of *H. capsulatum* cDNA

RNA was collected from log phase cultures of G217B using TRIzol Reagent (Invitrogen) following manufacturer's instructions. Contaminating DNA was removed by treatment with Amplification grade DNase I (Invitrogen). Reverse transcription reactions were carried out using SuperScript First-Strand Synthesis System (Invitrogen) following the manufacturer's protocol.

### Polymerase chain reaction

PCR amplification was performed using the TripleMaster high fidelity PCR system (Eppendorf, Westbury, NY) following the manufacturer's instructions. All primers were designed from sequences obtained from the *Histoplasma capsulatum* G217B genomic database (www.genome.wustl.edu) using MacVector sequence analysis software (Accelrys). Primers were synthesized by Integrated DNA Technologies (IDT, Coralville, IA).

### Overexpression of *HcDPPIVA* in *H. capsulatum*

We used an *H. capsulatum* expression vector pLBZ-1 described previously [12]. It carries a *PaURA5* marker for selection, an inverted telomeric region for linearization and maintenance, and cloning sites between the *H2B* 5′ and *CATB* 3′ flanking sequences. To overexpress *HcDPPIVA* in *H. capsulatum*, the *HcDPPIVA* full-length ORF was amplified from cDNA with primers (o-1) 5′-NNN**ggcgcgcc**ATGAAGGCATTTTCTCTT-3′ and (o-2) 5′-NNNNNN**cctgcagg**ttattacagatcttcttcagaaatcagtttttgttcAAATCTCAGGCCATTCCGATCC-3′ and cloned into pLBZ-1 using the *Asc*I and *Sbf*I sites (shown in bold type). A C-terminal c-myc tag was incorporated (underline); this tag was incidental for *H. capsulatum* expression in this study, but was used in *P. pastoris* expression as described below. The resulting plasmid was named pDPP-OE. Both pLBZ-1 (empty vector) and pDPP-OE were transformed into G217B*ura*5-23. Transformants were selected for uracil protrophy.

### Expression and purification of HcDppIVA in *Pichia pastoris*

Expression of *HcDPPIVA* in *Pichia pastoris* was performed using the EasySelect *Pichia* Expression Kit (Invitrogen). The *HcDPPIVA* ORF was amplified from cDNA with primers (p-1) 5′-NNNNNN**cacgtg**ATGAAGGCATTTTCTCT-3′ and (p-2) 5′-NNNNNN**gcggccgc**tcatcaatggtgatggtgatggtgAAATCTCAGGCCATTCCGATCC-3′ and cloned into pPICZA using the *Pml*I and *Not*I sites (shown in bold type). A carboxyl-terminal 6× His tag was also included (shown in underline). The resulting plasmid was named pDPP-PIC. *P. pastoris* was transformed according to the manufacturer's instructions. *Pichia* transformants were selected on 500 µg/mL zeocin. Methanol induction of expression was carried out according to manufacturer's instructions. We were unable to purify the recombinant protein using nickel affinity chromatography. Instead, HcDppIVA was purified from *Pichia* culture supernatant by the following protocol. *Pichia* culture supernatant was stirred with 1/10 vol of a 1 M Tris-HCl, pH 8.0 buffer and subjected to ammonium sulfate precipitation. Ballast proteins were precipitated from the solution at 30% saturation for 1 h at RT. The mixture was then centrifuged at 3500×*g* for 15 minutes and the collected supernatant was concentrated using a Vivaflow 200 unit (Sartorius AG, Goettingen, Germany) equipped with a hydrosart 10-kDa cut-off membrane. The resulting protein sample was subjected to column chromatography purification. All chromatographic separation steps were performed at room temperature on the high-performance liquid chromatography ÄKTA-Purifier 10 system (Amersham Biosciences AB, Uppsala, Sweden). All buffers used were filtered through 0.2 µm nylon membrane filters (Nalgene, Rochester, NY). The elution of proteins was monitored at a UV absorbance of 280 nm. Protein amounts were assessed using the BCA Protein Assay Kit (Pierce Biotechnology, Rockford, IL), with bovine serum albumin as a standard. The protein sample was chromatographically desalted on a 5-ml HiTrap™ Desalting column (Amersham) and then separated on an anion exchanger HiPrep™ 16/10 DEAE FF column (Amersham) equilibrated with 20 mM Tris/HCl (pH 8.0). Elution was carried out in 20 mM Tris/HCl (pH 8.0) amended with 1 M NaCl. Proteins were eluted in a linear salt gradient from 0 to 70% in 7 column volumes. DppIV active fractions eluted from the column at a conductivity range of 11–21 mS/cm were pooled together and concentrated using Vivaspin 20 units equipped with polyethersulfone 5-kDa nominal molecular mass cutoff limit membranes (Sartorius). The protein sample was then applied to gel filtration on a HighPrep™ 16/60 Sephacryl™ S-200 HR column (Amersham) equilibrated with PBS. Proteins were eluted at a flow rate of 1 ml/min in 1.5 column volume. Fractions containing DppIV activity were collected, concentrated and desalted on the HiTrap™ Desalting column, and subsequently separated in anion exchange chromatography on a MonoQ™ 5/50 GL column (Amersham) equilibrated with 20 mM bis-Tris/HCl (pH 6.5). Proteins were eluted in 10 column volumes in a linear salt gradient to 0.25 M NaCl. DppIV positive fractions were eluted at a conductivity range between 17 and 32 mS/cm. The final purification step involved gel filtration chromatography on a Superdex™ 200 10/300 GL column (Amersham) equilibrated with PBS. HcDppIVA was eluted in 1.5 column volumes in this buffer at a flow rate of 0.5 ml/min. Specific activity of the purified enzyme was calculated by enzyme assay using Gly-pro-AMC with reference to a standard curve determined using porcine kidney DppIV (Sigma).

### Determination of pH and temperature optima and effect on enzyme stability

To determine the pH optimum of recombinant HcDppIVA, enzyme assays using purified HcDppIVA with Gly-pro-AMC were performed in the following buffers: formate (pH 3), acetate (pH 4), piperazine (pH 5), MES (pH 6), PIPES (pH 7), HEPES (pH 8), and tricine (pH 9). Temperature optimum assays were performed in HEPES (pH 8) buffer at 4, 12, 25, 37, 42, 50 and 65°C. Stability was assessed by pre-incubating enzyme at each pH or temperature for 30 minutes and then performing an enzyme assay at pH 8 and 37°C to measure remaining activity. The incubation time for all enzyme assays was one hour.

### Truncation of substance P by HcDppIVA

5 mU recombinant HcDppIVA or 100 ng murine CD26 (R&D Systems) was incubated with 50 µg substance P (Sigma) in 50 mM Tris pH 8 at 37°C overnight. Reactions were then resuspended in 0.1% trifluoroacetic acid in 30% acetonitrile and separated using a Superdex Peptide 10/300 GL column (Amersham).

### Infection of mice with *H. capsulatum*

5–6 week-old female C57BL/6 mice (Harlan Laboratories, Madison WI) were inoculated intranasally with approximately 2×10^6^ yeast cells in a 20-µl volume. After one week, mice were euthanized by CO_2_ inhalation and lungs, livers, and spleens were harvested to evaluate CFU. Organs were placed in sterile distilled water and homogenized using a Stomacher 80 Biomaster (Seward, London UK). Homogenates were plated on Brain Heart Infusion agar plates and incubated at 22–24°C for 2–4 weeks.

## Results

### Purification and biochemical characterization of recombinant HcDppIVA

**Figure 1:**
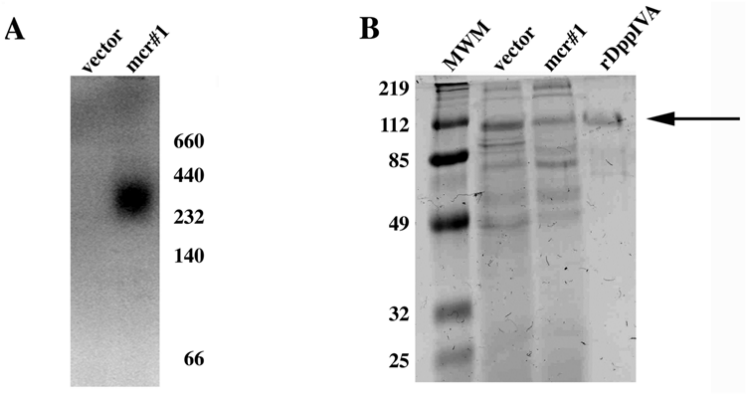
Expression and purification of recombinant HcDppIVA in *Pichia pastoris*. (A) Gly-pro-AMC zymogram of supernatants from *P. pastoris* transformed with empty vector and HcDppIVA-expressing *Pichia* (mcr#1). Molecular weights (kDa) are indicated on the right. (B) SDS-PAGE gel of supernatants from a non-expressing strain (vector) and mcr#1 as well as the purified fraction of HcDppIVA (rDppIV). The arrow indicates the band likely corresponding to the enzyme. The molecular weight markers (MWM) are in the first lane of the gel. The gel was stained with Coomassie Brilliant Blue.

**Figure 2:**
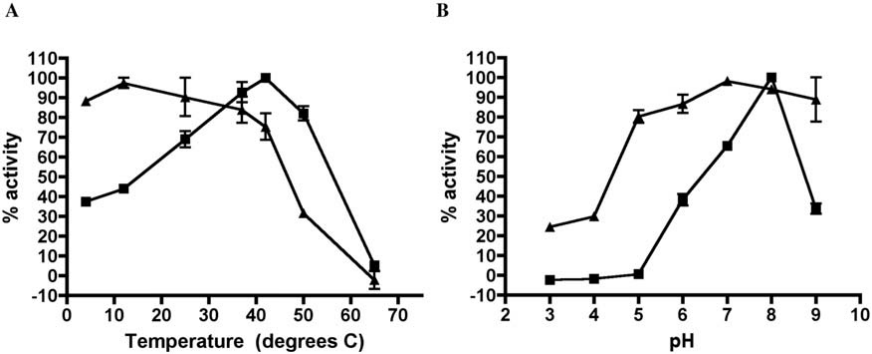
Activity and stability of purified recombinant HcDppIVA. A range of temperature (A) and pH (B) conditions were examined using substrate Gly-pro-AMC. To determine activity at different temperatures and pH, enzyme activity assays were performed at the indicated temperatures or pH values (squares). To determine stability, purified enzyme was incubated at the indicated condition for 30 minutes and subsequently assayed for remaining activity at pH 7, 37°C (triangles). The values in conditions with the highest activity were set at 100%. Error bars represent the variability between two experiments.

In order to test whether *HcDPPIVA* encoding a putative DppIV enzyme was functional, we expressed the *HcDPPIVA* open reading frame with its native signal sequence and a C-terminal histidine tag in the methylotrophic yeast *Pichia pastoris*. After transformation, we induced expression of *HcDPPIVA* by the addition of methanol to the medium. Culture supernatants were harvested and enzyme activity was detected using a zymogram assay. A fluorescent band appeared in *HcDPPIVA*-expressing *Pichia* supernatant but not a control strain transformed with empty vector, suggesting that *HcDPPIVA* encodes an active DppIV enzyme with a functional secretion signal (Figure 1A). The apparent molecular weight of the recombinant protein was larger than the predicted molecular weight of 88 kilodaltons, suggesting that the enzyme is assembled as a multimer and/or may be modified although we have not experimentally determined the basis for the size or whether multimerization or modification is necessary for function. Dimerization and/or glycosylation have been described for other DppIV enzymes, including *Porphyromonas gingivalis* DppIV [8] and human CD26 [13], [14]. To characterize the biochemical properties of HcDppIVA, we attempted to purify the C-terminal histidine-tagged enzyme using Ni^2+^ chromatography but were unsuccessful. Instead, we performed analytical-scale purification of recombinant enzyme from *Pichia pastoris* culture supernatant using anion exchange and size exclusion chromatography (Figure 1B). We measured DppIV activity of the purified preparation towards Gly-pro-AMC in different buffers and temperatures to determine optimal enzymatic conditions and stability of the enzyme. Similar to reported values for other DppIV enzymes, HcDppIVA is optimally active at 42°C and pH 8. Additionally, HcDppIVA is destabilized by low pH or high temperature (Figure 2). These data indicate that *HcDPPIVA* encodes a DppIV-type enzyme with biochemical properties similar to those previously reported for DppIV enzymes. As further demonstration of recombinant *H. capsulatum* enzyme expression in *P. pastoris*, we replaced the carboxyl-terminal his-tag with a c-myc epitope tag followed by transformation, expression, and supernatant isolation as described above. Western immunoblotting with anti-c-myc antibody revealed a reactive band from *P. pastoris* transformed with the expression plasmid but not empty vector (data not shown). Furthermore, immunoprecipitation of this recombinant *P. pastoris* supernatant with anti-c-myc antibody yielded DppIV activity (data not shown).

### HcDppIVA can cleave a host immunoregulatory peptide

**Figure 3:**
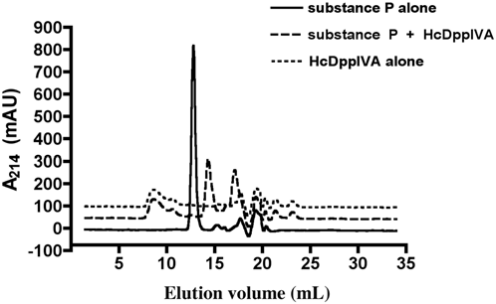
Chromatogram showing substance P truncation by HcDppIVA. 50 µg substance P was incubated with or without 5 mU recombinant HcDppIVA. Solid line: undigested substance P; heavy dotted line: substance P + HcDppIVA, light dotted line: purified HcDppIVA alone. Elution volumes for truncated substance P products correspond to those of CD26-mediated truncation (data not shown).

To determine whether HcDppIVA can cleave the host immunoregulatory peptide substance P, we incubated rHcDppIVA or CD26 with 50 µg substance P in 50 mM Tris pH 8 at 37°. Reactions were then resuspended in 0.1% trifluoroacetic acid in 30% acetonitrile and separated using a Superdex Peptide 10/300 GL column. We observed a single peak with retention volume of 12.8 mL when substance P was incubated without enzyme. After incubation with rHcDppIVA or CD26, two peaks were detected with retention volumes of 14.3 mL and 17 mL indicating that substance P was cleaved (Figure 3). CD26 cleaves substance P after the proline in the second position from the amino-terminus, releasing Arg-Pro and then subsequently cleaves again releasing Lys-Pro [15], [16]. We have not identified the substance P products after HcDppIVA or CD26 mediated cleavage in this study, but it is likely that the first peak represents the product of the first cleavage event (SP 3-11), while the second is the product after removal of all four amino acids (SP 5-11). The similar elution profiles after incubation with both enzymes indicates that HcDppIVA can cleave substance P in the same manner as CD26.

### Overexpression of *HcDPPIVA* in *H. capsulatum*

**Figure 4:**
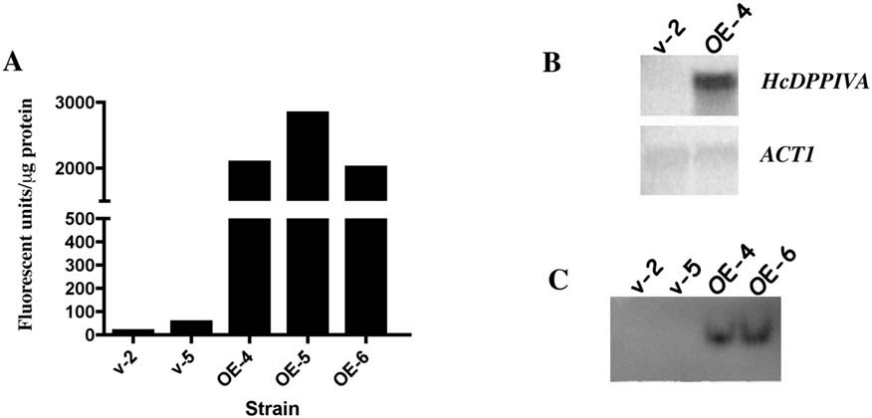
Overexpression of *HcDPPIVA* in *Histoplasma capsulatum*. (A) DppIV activity using substrate Gly-pro-AMC in supernatants of G217B*ura*5-23 strains carrying the empty vector pLBZ-1 (v-2, v-5) and strains carrying the pDPP-OE overexpression plasmid (OE-4, OE-5, OE-6). (B) Northern analysis using *HcDPPIVA* or *ACT1* specific hybridization probes. (C) Gly-pro-AMC zymogram using supernatants of indicated strains.

In order to manipulate expression of *HcDPPIVA* in its native organism, we overexpressed it in *H. capsulatum* strain G217B*ura*5-23 by placing the open reading frame under the control of the heterologous *H2B* promoter and *CATB* terminator sequences on a multicopy plasmid. We observed a substantial increase in transcript levels (figure 4B) and secreted DppIV activity in culture supernatants when *H. capsulatum* was transformed with this *HcDPPIVA* expression construct. This activity increase was monitored quantitatively by enzyme assay (figure 4A) and qualitatively by zymography (figure 4C). These data indicate that lack of expression from native *HcDPPIVA* regulatory sequences is responsible for the previously reported lack of detectable secreted activity under standard laboratory conditions (Cooper *et al.*, submitted). When expression of this gene is increased using heterologous regulatory sequences, HcDppIVA is produced and secreted into culture supernatants.

### Evaluation of an *HcDPPIVA*-null mutant in virulence

We previously constructed an *HcDPPIVA*-deletion mutant using a Ku-deficient strain of *H. capsulatum*. Consistent with the present study, no detectable differences were observed in secreted activity due to loss of the gene, further indicating a lack of expression under these conditions (Cooper *et al.*, submitted). However, it is possible that *HcDPPIVA* expression is induced during infection. In order to determine whether *HcDPPIVA* is important for pathogenesis, we used a mouse model of histoplasmosis as previously described [17]. We did not observe a virulence defect due to *HcDPPIVA* deficiency relative to the parental Δ*KU70/KU80* strain based on measurement of fungal burden in lungs, liver, and spleen one week after intranasal infection (data not shown). We cannot exclude an *HcDPPIVA* deficiency-associated virulence defect that might be detected in survival, histopathology, or other in vivo or in vitro infection models.

## Discussion

The opportunistic fungal pathogen *Aspergillus fumigatus* secretes a DppIV enzyme that has been postulated as a virulence determinant [18]. This assessment is partly based on the observation that AfDppIV cleaves host immunoregulatory peptides *in vitro* [19]. Additionally, pre-treatment of rabbits with recombinant AfDppIV reduces inflammation caused by histamine, possibly by the inactivation of neuropeptides and other immunomodulatory peptides [20]. The closest homolog to *A. fumigatus DPPIV* in *H. capsulatum* is *HcDPPIVA*. *HcDPPIVA* encodes a putative DppIV enzyme that contains a predicted secretion signal. However our previous study showed that under standard *in vitro* culture conditions *HcDPPIVA* did not detectably contribute to secreted DppIV activity in *H. capsulatum* culture supernatant (Cooper *et al.*, submitted). This study provides evidence indicating that *HcDPPIVA* encodes an active DppIV enzyme that is capable of being secreted. First, HcDppIVA contains an N-terminal signal sequence that functions properly in *Pichia pastoris* for secretion of the protein into the culture supernatant. Second, overexpression of *HcDPPIVA* in *H. capsulatum* leads to increases in the level of DppIV activity in the culture supernatant. Together, these data indicate that *HcDPPIVA* encodes a fully functional DppIV enzyme capable of being secreted by *H. capsulatum*.

Although under standard laboratory conditions *HcDPPIVA* does not appear to contribute to the secreted DppIV activity, it is possible that this gene is expressed only during a specific growth condition such as during infection. It is not unusual for members of a single protease family to be differentially expressed, as demonstrated in a number of fungal systems. In *H. capsulatum*, different serine protease activities are produced at different culture growth stages [21]. Another example is the *SAP* family of *C. albicans* protease genes with 10 currently identified members. Under standard laboratory conditions, Sap2 is the major protease to be expressed [22]. However the other *SAP*s are upregulated in a wide variety of conditions such as during yeast to hyphal transition, biofilm formation, phenotypic switching, or while infecting a host (for review see [23]). Secreted dipeptidyl and tripeptidyl petidase homologs are upregulated during infection by dermatophyte fungi of the genus *Trichophyton* [24] and the dimorphic fungal entomopathogen *Beauveria bassiana* [25].

We initially hypothesized that HcDppIVA influences infection by cleaving and thereby altering the activity of host immunomodulatory chemokines such as substance P, a mechanism demonstrated for *Poryphyromonas gingivalis* [7], [8] and postulated for *Aspergillus fumigatus* [18]–[20]. Our results indicate that *HcDPPIVA* is not required for virulence of *H. capsulatum* in a mouse model of histoplasmosis assessed by fungal CFU burden in infected tissues. However, an effect of DppIV absence may be revealed by histopathological analysis of *H. capsulatum*-induced granulomas or measurement of chemokine activity. Furthermore, because *HcDPPIVB* also can provide secreted DppIV activity, it is possible this gene contributes during infection and may compensate in the absence of *HcDPPIVA*.

Because both HcDppIVA and HcDppIVB are capable of being secreted and at least one can cleave the host immunoregulatory peptide substance P, a further investigation into DppIV enzymes as potential virulence factors of *H. capsulatum* is warranted. Substance P is a neuropeptide that also has an important role in immune regulation, one of which is to upregulate interferon-gamma production [26]. This peptide binds its receptor NK-1 with high affinity and elicits a pro-inflammatory response. N-terminal truncation of substance P destabilizes the peptide leading to its rapid degradation by aminopeptidase M [15], [16]. Additionally, the N-terminal prolines in substance P prevent binding to the other tachykinin receptors NK-2 and NK-3 [27]. Removal of these prolines presumably could lead to loss of NK-1 specificity, and therefore shift the relative binding to include NK-2 and NK-3. Pro-inflammatory signaling mediated by substance P acts through NK-1 [28]–[30], indicating that a shift away from NK-1 receptor engagement may reduce overall inflammation. Other potential host DppIV substrates with a role in inflammation include such peptides as neuropeptide Y and vasoactive intestinal peptide [31], [32] as well as chemokines such as RANTES and eotaxin [33], [34]. Further experiments are planned to test substrate specificity and relative activity of *H. capsulatum* DppIV enzymes in comparison to those of CD26 and to determine possible physiological effect during infection.
